# Assessment of dietary diversity and associated factors among lactating mothers in Debub Bench District

**DOI:** 10.1016/j.heliyon.2021.e07769

**Published:** 2021-08-14

**Authors:** Dinaol Abdissa Fufa, Teshale Darebo Laloto

**Affiliations:** Department of Nutrition and Reproductive Health, School of Public Health, College of Health and Medical Sciences, Mizan-Aman, Mizan-Tepi University, Ethiopia

**Keywords:** Minimum dietary diversity, Lactating mother, Debub bench, Ethiopia

## Abstract

**Objective:**

To identify dietary diversity and its associated factors among lactating women in the Debub Bench district.

**Methods:**

Cross-sectional study design was conducted among 836 lactating women from January 1st to March 31st, 2019, in Debub Bench district. The outcome variable of the study was determined based on the proportion of lactating mothers who fed less than five major food groups to mothers who fed more than five major food groups out of nine (9) food groups. Lactating mothers who fed less than five of the major food groups were categorized under unacceptable dietary diversity. Data were first collected through face-to-face interviews by validated structured questionerers and then entered in Epi-data version 4.6.0.2 software. A bivariate and multivariate logistic regression analysis were later conducted using IBM SPSS version 26 software. During the analysis, multicollinearity was check by using the tolerance test and variance inflation factors (VIF), Hosmer-Lemeshow goodness of fit test was used to see model fitness, and adjusted odds ratios and their 95% confidence interval at P values ≤0.05 were considered to determine statistically significant factors.

**Result:**

A total of 836 lactating mothers had participated in the study. The response rate was 91.26%. The mean age of the participants was 29 years (SD ± 6.7). The study found that the magnitude of unacceptable dietary diversity score was 72.4% (95% CI: 69.5–75.5). The study also found that factors such as nutrition information (OR = 4, 95% CI: 2.64–6.08), absence of garden (OR = 2.35, 95% CI: 1.19–4.61), absence of latrine (OR = 6.86, 95% CI: 3.26–14.56) and household food insecurity (OR = 5.23, 95% CI: 3.64–7.46) were significantly associated with unacceptable dietary diversity.

**Conclusion:**

The finding of this study showed that information about nutrition, absence of latrine, absence of garden, and household food insecurity were significantly associated with dietary diversity. Based on the finding of the study, the following recommendations are made. First, strategies and programs targeted towards promoting dietary diversity and good health among lactating women should be made at all levels. Second, lactating mothers should be adequately provided with nutritional information. Three, mothers should be empowered to alleviate household food insecurity by leveraging their premises for gardening diversified and nutritious vegetables.

## 1 Introduction

Studies after studies indicated that during lactation and breastfeeding, the nutrition status of mothers is among the essential to closely follow and care about. Among others, on one hand, this is because the period of lactation and breastfeeding is the time when the mother exhibit high physiologic demand for nutritionally ritch food items in order to gain extra energy. During lactation, extra energy is required because lactating mothers produce approximately 700–800 ml of milk per day. On the other hand, it is because the nutritional status of a lactating mother is directly related with the nutritional status and health condition of infants and young children [1, 2, 3].

It is indisputable the at maternal poor nutrition has an impact on maternal health and survival of the mother and the unborn child. For instance, anemia resulting from nutrient deficiencies such as iron and folic acid is a significant risk factor for hemorrhage that could ultimately lead to maternal mortality [4].

Dietary diversity is the state of consuming different food groups within twenty four hours of a given day [5]. According to a 2010 guideline for measuring household and individual dietary diversity, women who consume less than four food groups are consider as having low dietary diversity. Whereas, women who consume for or five food groups are consider as having medium dietary diversity. Whereas, women who consume more than five food groups out of the nine food groups considered as having high dietary diversity [5].

In general, women in reproductive age groups need more energy than others, but the energy requirements during lactation increase by 42% as compared to pregnant and other women in reproductive age group [1]. In a developing country, lactating women are more susceptible to malnutrition due to physiological and hormonal changes. The inability to meet the physiological demands for nutrients among lactating mothers may result in malnutrition [6].

In Ethiopia, the magnitude of low dietary diversity ranges between 25.9% and 56.4% [7, 8]. In developing countries, the dominant food group is a starchy food which is low in protein and has low rate absorption [9, 10].

The study area is a cash cropping area where diet depends on staple, starch, fruits, and routs. Therefore, this study aimed to identify dietary diversity and associated factors among lactating mothers in Debub Bench districts.

## 2 Method

### 2.1 Study area, design, and period

A community-based cross-sectional study was done from January 1^st^ to March 31^st,^ 2019, at Debub Bench district (woreda). This district is one of the eight woredas in Bench Sheko Zone, South Nation Nationalities, and Peoples Regional State in Ethiopia. Debub Bench district is found between Meinit Shasha, Gurafarda, Sheko, Semien Bench, She Bench, and Meinit Goldiya districs in the southwest, north, northeast, east, and southeast respectively. Based on the Ethiopian Census - 2017 Projection, this district has a total population of 142,940, of which 69,945 were men and 72,995 were women. 9,956 or 6.97% of its population were urban dwellers [11]. The majority of the population (60.7%) were protestants, orthodox christians were 20.01%, muslim were 6.1%, and tose who practice traditional religious belief were 13.2%. The district consists of 25 rural kebeles and one township administration. Regarding health care service coverage, there were 31 health posts and four health centers. The entire expected lactating mothers in the nominated kebele were 5,496.

### 2.2 Source and study population

**Source population**: Lactating women who lived more than six months in the current study area. **Study population**: All randomly selected lactating mothers in selected kebele.

**Inclusion criteria:** Lactating women who lived at least six months and above in the current study area.

**Exclusion Criteria:** Lactating women who were sick or unable to communicate during data collection.

### 2.3 Sample size calculation

The required number of samples for the present study was calculated using STAT CALC application of EPI- INFO version 7 software. The sample size is calculated using the double population proportion formula with the assumption: Two-sided confidence level at 95%, Power at 80%, and 0.5 margins of error, 1.5 design effect, and 5% of contingency non-response rate. Table 1.

**Table 1:** Sample size determination by using factors associated with dietary diversity among Lactating Mothers in Debub Bench district, Ethiopia, 2019.

Variables	Confidence level	Power	% of controls exposed	% of cases with exposure	Adjusted OR	Sample size for both group
Drinking Water	95%	80	2	7.2	3.8	582
Age of Lactating Mother 20–24 years old	95%	80	5.27	16.83	3.63	264
Occupational Status of the Mother	95%	80	7.28	22.2	3.63	202

The exposed variables were the source of drinking water, age of lactating mother, and occupational status of the mother [7, 8]. Finally the large sample size was taken (582∗1.5 = 873 ∗0.05 = 43.65 = 873 + 43.65 = 916).

### 2.4 Sampling procedures

From the total of 8 districts in the Bench-Shako zone, Debub Bench was select by simple random sampling. Similarly, from the total of 25 kebeles, in Debub Bench, 11 kebeles were selected using simple random sampling techniques. Census was done in selected kebeles to list the sampling frame. Before starting data collection, the households having lactating women were identified by strictly following eligibility criteria. The households that only fulfilled the inclusion criteria were considered as study unite. The current study's sample size was proportionately allocated to each selected kebele.

We used the simple random sampling method, to select the first sampling unit. The remaining were selected systematically at an interval of K. Interval (K value = 6) was determined for each selected kebeles by dividing the total eligible lactating mother in the kebele to the sample size. We had randomly chosen one mother whenever we encountered more than one eligible lactating mother in a given household.

### 2.5 Data collection procedures and instruments

The data were collected by six trained experts who hold BSC degree in Public Health. A one-day training was given to data collectors regarding anthropometric measurements, interviews, selection principles of study unit, and approach to respondents before starting the data collection. Data were collected with a home-to-home visit through anthropometric measurement and direct interviewing of eligible subjects by a pre-tested organized questionnaire. The questionnaire was translated from the English language into the local language, “Amharic.” The translation was carried out by a language expert. To test the correctness of the Amharic version of the questionnaire, the Amharic version of the questionnaire was also translated back to the English language by an individual who was blind to the original version and fluent in English and local languages. The questionnaire includes questions about socio-demographic characteristics, Household Wealth Index, Women's Dietary Diversity Score (DDS), and nutritional status of a lactating mother. The Household Wealth Index was assessed by the scoring of Household Wealth Index factors based on the number and kinds of productive assets participants had. Asset may refers to domestic animals; vehicle; the characteristic of housing like flooring material, latrine facility, source of drinking water, and home environment [12]. The household food security level was assessed by nine standard questions of the Household Food Insecurity Access Scale (HFIAs) developed by Food and Nutrition Technical Assistance (FANTA) in 2007. Each respondent was asked about food eaten and food shortage, causing the respondents not to eat the whole day or eat at night only in the past one month (4 weeks) earlier to the survey. All yes responses were coded as one while and no response were coded as zero. Finally, all responses were summed to yield the household food insecurity index. The index had internal consistency (Cronbach alpha ¼ 0.90). Then, give zero code for food insecurity, and give one code for food security [13]. Women's Minimum Dietary Diversity (MDDs) status was determined based on the proportion of lactating mothers who fed less than five major food groups to lactating mothers who fed five and more major food groups out of nine food groups. The lactating mothers who feed less than five the major food group were considered as having unacceptable (low) minimum dietary diversity [5].

### 2.6 Anthropometric measurement

Middle-upper Arm Circumference: the middle-upper arm circumference of lactating mothers was measured by the MUAC tape made from synthetic paper (model No; S0145620, which UNICEF manufactured for this purpose). Before starting measuring middle-upper arm circumference of lactating mothers, we removed any clothing that may cover the lactating mother's left arm then calculated the midpoint of the lactating mother's left upper arm by first locating the tip of the lactating mother's shoulder, bent the lactating mother's elbow at the right angle, and inspected the tension of the tape on the lactating mother's arm. We also made sure that the tape has proper tension and was not too tight or too loose. We read and called out the measurement to the nearest 0.1cm [14] when the tape was in the correct position on the arm with the correct tension.

BMI was ground on a weight-to-height ratio considered a good index of body fat and protein stores. BMI is (weight (in kilograms)/height (in meters)^2^) weight (in kilograms) divided by the height (in meters^2^). The Categories of Body Mass Index are: Normal ≥ 18.5, Grade I 17.0–18.49, Grade II 16.0–16.99, and Grade III <16 [14].

### 2.7 Data quality control

To ensure the quality of the data the questionnaire was pre-tested on 5% of the study sample among not selected kebele. A one-day training was also given for data collectors on the instruments, method of data collection, how to take anthropometric measurements, ethical issues, and the purpose of the study. During training, to minimize the intra and inter-observer's variability and random anthropometric measurement error, the data collector's relative technical error of measurement (%TEM) was calculated based on data gathered from ten lactating mothers. The level of putative relative technical errors measurement for intra-observers was less than 1.5%, whereas inter-observers were less than 2%. Data collectors' accuracy status of anthropometric measurements (MUAC, weight, and height) was standardized with their trainer during training and pretesting [15]. Data gatherers measured at least 2 (two) separate MUAC and height (anthropometric measurements) of lactating women and documented the averages. Double data entry was also done to compare two data cells and resolve whenever there was some difference.

### 2.8 Data processing and analysis

The collected information (data) was checked daily for completeness and cleaned for missed and implausible values accordingly. Then each completed and cleaned questioner was assigned a unique code, double entered into Epi Data version 4.6.02 software and later exported to IBM SPSS version 26 software for analysis.

Descriptive analyses was done to explore the socio-economic characteristics of the respondents, and results were presented by using frequency tables.

The outcome variable was recoded to dichotomous outcomes as “unacceptable dietary diversity” and “good dietary diversity”. Those with low dietary diversity were coded “1” and poor dietary diversities were coded “0”. The independent variables were coded based on former related published articles and responses in the data [8,16, 17].

The family wealth index data was constructed using principal component analysis method, and then we computed the composite score. Finally, the household wealth quintiles compiled by assigning the household score to the respective household, then ranking each household in the community by their score, and allocating the distribution into three equal categories; poor, middle, and rich.

To measure household food security position, the Household Food Insecurity Access Scale was used to classify whether the households were food insecure or not.

Women's Minimum Dietary Diversity (MDDs) status was determined based on the proportion of lactating mothers who fed less than five major food groups to lactating mothers who fed five and more major food groups out of nine food groups. The lactating women who fed less than five of the major food group were deemed as having unacceptable minimum dietary diversity.

The multicollinearity effect was checked by looking at the standard error. Only non-collinear covariates were included in binary logistic regression to assess the possible association between each independent and dependent variable. Only those variables with a P-value of ≤0.25 during bivariate analyses were chosen for subsequent analyses using multiple logistic regressions to control all possible confounders and detect variables significantly associated with the outcome variable. The model fitness of the study was tested by Hosmer-Lemeshow goodness of fit test. In this study, AOR (adjusted odds ratios) and 95% confidence interval (CI) of the association were declared statistical significance at P values ≤0.05.

### Ethical approval

This study was done based on the declaration of Helsinki. Ethical clearance was also obtained from Mizan-Tepi University Institutional Health Research Ethics Review Committee (IHRERC) IRB/012/20. A permission letter was obtained from Mizan-Tepi University and submitted to the Debub Bench District health office. Participants were first informed about the purpose of the study, procedure and duration, possible risks, and benefits. Only those participants who volunteered and signed on an informed concent form were interviewed.

## 3 Result

### 3.1 Socio-demographic characteristics of the respondents

A total number of 836 lactating women had joined the current study with a response rate of 91.26%. The mean ± S.D. (standard deviation) of the lactating women's age was 29 ± 6.7 years old. More than three-fourths (76%) were protestant, 94.2% were rural dwellers, 97.8% were married, and 91% were housewives. Table 2.

**Table 2:** Socio-demographic characteristics of study the respondents in Debub Bench district, Ethiopia, 2019.

Variable	Frequency	Percentage
**Age category**		
18–29	354	42.3
30–39	298	35.7
40–49	184	22
**Family size**		
Five and above	215	25.78
Below five	621	74.22
**Source of drinking water**		
Tap water	669	80
Pumping water	146	17.5
Protected well	21	2.5
**Presence of Latrine**		
Yes	730	87.3
No	106	12.7
**Home gardening practice**		
Yes	612	73.2
No	224	26.8
**Maternal educational status**		
Have formal education	202	24.2
Have no formal education	634	75.8
**Husband educational status**		
Have formal education	272	32.5
Have no formal education	564	67.5
**Husband occupation**		
Government employee	187	22.4
Merchant	100	12
Farmer	435	52
Daily laborer	114	13.6
**Household wealth index**		
Poor	294	35.2
Medium	419	50.1
Rich	123	14.7

### 3.2 Maternal health care and feeding practice

More than one-third of lactating mothers, 297 (35.5 %), had not received postnatal care (PNC) in the previous labor. Table 3.

**Table 3:** Maternal Health Care and Feeding Practice in Debub Bench district, Ethiopia, 2019.

Variable	Frequency	Percentage
**Attending ANC for current child**		
No	52	6.2
Yes	784	93.8
**Attending PNC for current child**		
No	110	13.16
Yes	726	86.84
**Place of delivery**		
Health Center	330	39.5
Hospital	401	47.9
Home	105	12.6
**Avoid eating any food during lactation**		
No	536	64.12
Yes	300	35.88
**Eat any additional foods during lactation**		
No	649	77.64
Yes	187	22.36
**Change in food intake during lactation**		
No change	613	73.27
Frequency of meal	120	14.43
Amount of meal	103	12.3
**Number of meals/day**		
3 meals	526	62.9
≥3 meals	310	37.1

### 3.3 Household food security status

The household's overall food security position was 67.8%, (95%, CI: 64.8–70.9) for food-insecure households and 32.2%, (95%, CI: 29.1–35.2) for food secure households.

### 3.4 Nutritional status related factors

The mean ± S.D weight and height of the study subjects were 55 ± 3.5 kg and 1.67 ± 0.23m, respectively. The mean BMI of the lactating women was 19.3(SD = ± 2.57 kg/m^2^) and the overall magnitude of maternal underweight was 35.62% (95% CI: 33.1–37.2).

### 3.5 Dietary diversity score of lactating mother

**Figure 1:**
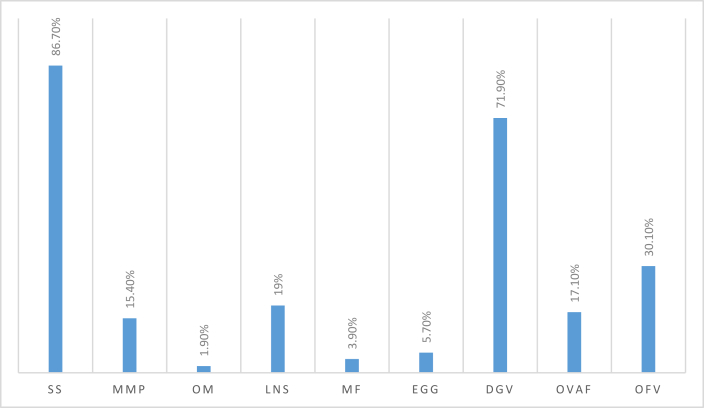
Percentage of food groups consumed by respondents in the past 24 h, among lactating mothers live in Debub Bench district southern Ethiopia 2019. Notes: Starchy staples (S.S.), Milk and milk products (M.P.), Organ meat (O.M.), Legumes, nuts and seeds (LNS), Meat and fish (M.F.), Dark green leafy vegetable (DGV), Other Vitamin A-rich fruits and vegetables (OVAF), and Other fruits and vegetables (OFV).

The entire unacceptable minimum dietary diversity score (<5 food groups) among the study participant were 72.4%, (95% CI: 69.5–75.5) during the previous 24 h of the survey. The majority (86.7%) of the lactating mother had consumed the Starchy staples food group followed by dark green leafy vegetable (71.9%) during 24 h preceeding data collection time. The least consumed food group was Organ meat (1.9%). Figure 1.

### 3.6 Factors associated with maternal dietary diversity score

In multiple logistic regression, the factors associated at p-value 0.05 with unacceptable minimum dietary diversity among lactating women were: having no nutrition information, having no garden, absence of latrine, and food insecurity status were. In the present study, respondents' nutrition information was found significantly associated with unacceptable minimum dietary diversity. Those individuals who had not nutrition information were four times more likely to have unacceptable minimum dietary diversity than those individuals who had nutrition information (AOR = 4, 95% CI: 2.64–6.08). Lactating women who had no garden were two times more likely to have unacceptable minimum dietary diversity as compared to their counterpart (AOR = 2.35, 95% CI: 1.19–4.61). Lactating women from the food-insecure household were five times more likely to have unacceptable minimum dietary diversity as compared with lactating mothers from food secured household (AOR = 5.23, 95% CI: 3.64–7.46). Mothers who had no latrine were near to seven times more likely to have unacceptable minimum dietary diversity as compared with those had latrine (AOR = 6.86, 95% CI: 3.26–14.56). Table 4.

**Table 4:** Bivariate and multivariable logistic analysis showed factors associated with unacceptable minimum dietary diversity among lactating women in Debub Bench district, Ethiopia, 2019.

Associated factors	Category	Low dietary diversity		OR (95% CI) (Crude)	P-values (Crude)	OR (95% CI) (Adjusted)	P-values Adjusted
		<5 food group	≥5 food group				
Have nutritional information	No	415	86	3.68 (2.68–5.0)	0.001	4 (2.64–6.08)	0.002
	Yes	190	145	1		1	
Paternal educational status	Have no formal education	402	162	0.84 (0.6–1.17)	0.24	0.55 (0.32–1.2)	0.1
	Have formal education	203	69	1		1	
Have garden	No	180	44	1.8 (1.24–2.61)	0.002	2.35 (1.19–4.61)	0.0001
	Yes	425	187	1		1	
Presence of latrine	No	544	201	1.3 (0.85–2.12)	0.22	6.86 (3.23–14.5)	0.01
	Yes	61	30	1		1	
Household food security status	Insecure	467	100	4.4 (3.21–6.11)	0.0001	5.23 (3.67–7.46)	0.001
	Secured	138	131	1		1	
Household wealth index	Poor	199	95	0.86 (0.54–1.37)	0.54	0.57 (0.34–1.17)	0.1
	Medium	319	100	1.3 (0.84–2.06)	0.225	1.1 (0.65–1.97)	0.2
	Rich	87	36	1		1	

CI = Confidence Interval, OR = Odds Ratio.

## 4 Discussion

Unacceptable minimum dietary diversity among lactating mothers is a potential cause for maternal undernutrition. Relatedly, Anemia resulting from nutrient deficiencies such as iron and folic acid is a significant risk factor for hemorrhage that could lead to maternal mortality. This study has determined the magnitudes of unacceptable minimum dietary diversity and associated factors among lactating mothers living in Debub Bench district, Ethiopia. The respondent's prevalence of unaccepted minimum dietary diversity was 72.4% (95% CI: 69.2–75.4). This finding is in line with a study conducted in a peri-urban area of Nepal [17]. Moreover, higher than the study in Angacha districts Southern Ethiopia, Debre Tabor, Ethiopia, and the study conducted in three countries (Bangladesh, Vietnam, and Ethiopia) [7, 16, 18]. This variation could be due to the difference in the sample size, the study period, seasonal variation, and another possible explanation is food security status change. On the contrary, most previous studies had a lower prevalence of food insecurity status than the present study.

In the present study, lactating mother who had no nutritional information, lactating mothers from households that have no garden, household food insecurity, and absence of latrine were significantly associated with minimum dietary diversity. Lactating mothers who had nutrition information met minimum acceptable dietary diversity in their daily diet compared to those with no nutrition information. This finding aligns with other evidence in Sidama, Ethiopia and (Bangladesh, Vietnam, and Ethiopia) [18, 19], this might be because nutritional information can increase the Knowledge of lactating mothers regarding nutrition and the importance of varieties of foods, and the lactating mother may have taken these varieties of food groups.

A lactating mother from a household not having a home garden was two times more likely to have unacceptable minimum dietary diversity than lactating mothers from a household with a garden. The current finding is similar to the findings conducted in Sidama and Tigray, Northern Ethiopia [18, 19]. The similarity might be due to lactating mothers who demonstrate home gardens would grow different kinds of vegetables and fruits and benefits from the home garden to diversify their daily food intake and may obtain additional diet options that enhance the diversity of the lactating mothers' food consumption of different food groups in their diets.

A lactating mother from a household with no latrine was nearly seven times more likely to have unacceptable minimum dietary diversity than lactating mothers from a household with a latrine. We have not found evidence supporting this study, but the possible reason might be open defecation results in an increased risk of diarrheal and other illness, which in turn causes difficulty of eating and might contribute to the low dietary diversity during the study period.

Finally, a lactating mother from food insecure household was five times more likely to have unacceptable minimum dietary diversity when compared with those lactating mothers from food secure household. The current study is in line with a study done in Angecha, southern Ethiopia [16]; this could result from that household food security enhance lactating mother, the intake of adequate quantity and quality of diet that contributes to having a better minimum acceptable dietary diversity score and nutritional status of a lactating mother. Unlike other studies, in the current study, some factors (source of drinking water, age of lactating mother, and occupational status of lactating mothers) were not significantly associated with minimum dietary diversity.

Besides, due to the nature of the cross-sectional study design, it is not easy to establish the cause-effect relationship between the predictors and the outcome variable. Furthermore, recall bias also potential limitations that might have affected the accuracy of the information, especially related to 24 h dietary group recall.

## 5 Conclusions

The finding of this study showed that information about nutrition, absence of latrine, absence of garden, and household food insecurity were significantly associated with dietary diversity. Based on the finding of the study, the following recommendations are made. First, strategies and programs targeted towards promoting dietary diversity and good health among lactating women should be made at all levels. Second, lactating mothers should be adequately provided with nutritional information. Three, mothers should be empowered to alleviate household food insecurity by leveraging their premises for gardening diversified and nutritious vegetables.

## Declarations

### Author contribution statement

Dinaol Abdissa Fufa, Teshale Darebo Laloto: Conceived and designed the experiments; Performed the experiments; Analyzed and interpreted the data; Contributed reagents, materials, analysis tools or data; Wrote the paper.

### Funding statement

This research did not receive any specific grant from funding agencies in the public, commercial, or not-for-profit sectors.

### Data availability statement

Data will be made available on request.

### Declaration of interests statement

The authors declare no conflict of interest.

### Additional information

No additional information is available for this paper.
